# Supplementary material for: Temporal interference stimulation disrupts spike timing in the primate brain
Source: Nat Commun. 2024 May 29;15:4558. doi: 10.1038/s41467-024-48962-2 (PMC11137077; doi:10.1038/s41467-024-48962-2)
Supplement: Supplementary file 1 — Supplementary Information [file 41467_2024_48962_MOESM1_ESM.pdf]

**Supplementary Information for**

# Temporal interference stimulation disrupts spike timing in the primate brain

Pedro G. Vieira[1☯], Matthew R. Krause[1☯*], Christopher C. Pack[1]

1. Montreal Neurological Institute, McGill University
Montreal, Quebec, Canada
☯ These authors contributed equally.
* Corresponding Author: matthew.krause@mcgill.ca

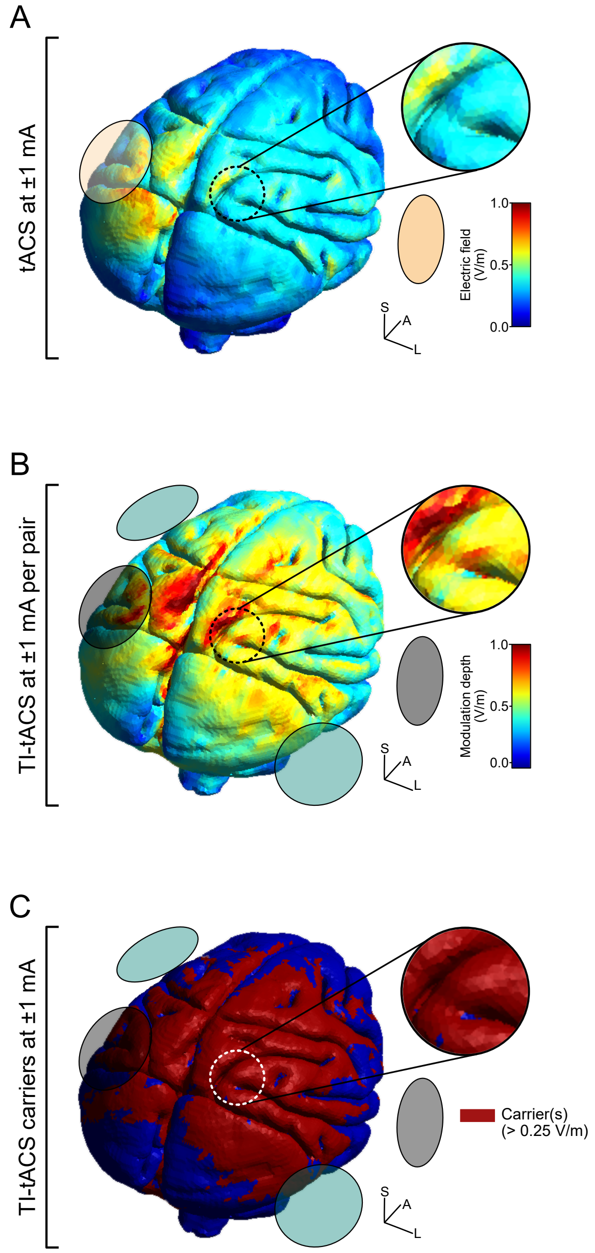


**Supplementary Figure 1: Whole-brain maps of stimulation effects. A** Electric field produced by our 7A/MT conventional tACS montage at ±1 mA of current. The same montage was also used for AM-tACS experiments. **B** Modulation depth produced during TI-tACS targeted at 7A/MT. This condition used both the electrodes in A (yellow/black) as well as a second pair (teal). ±1 mA current was delivered through each pair. **C** TI-tACS carriers individually spread over larger swathes of the brain than amplitude modulation. Red indicates locations where at least one carrier produces an electric field of 0.25 V/m or more, a level that has been shown to affect neural activity in lower-frequency conventional tACS experiments[3,4]. L: Lateral, A: Anterior, S: Superior. Dotted circles indicate the location of the recording chamber. Fields within the chamber (i.e., near our recording sites) are shown enlarged as insets.

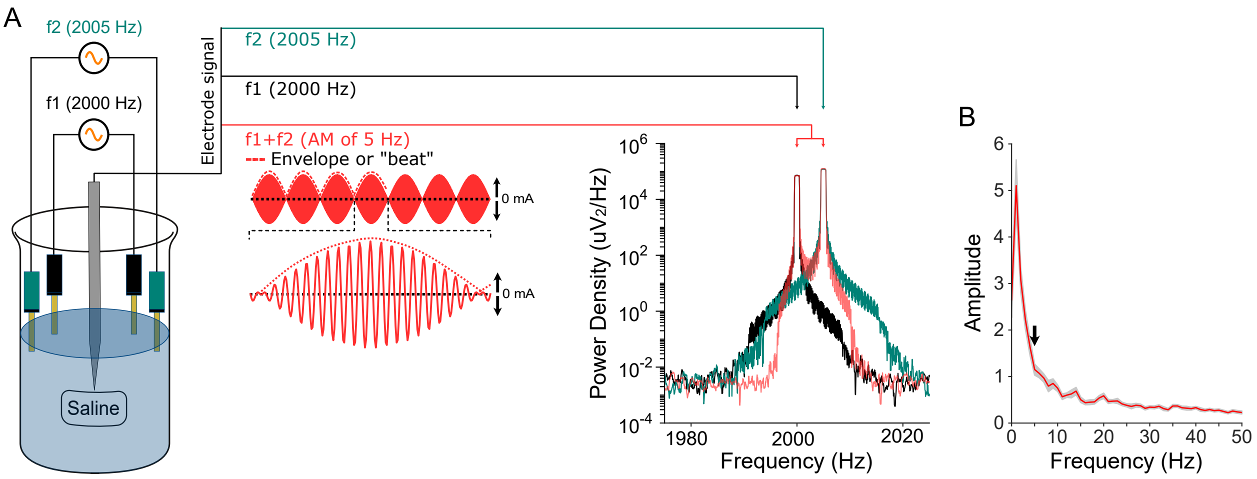


**Supplementary Figure 2: Validation experiment. A** Recordings from a saline bath confirm that no low-frequency artifacts, especially at the AM frequency (red, inset arrow), were produced by our stimulation and recording systems. Only components at the two carrier frequencies (green and black) were present in these data. **B** Importantly, no power could be detected at the beat frequency (red) suggesting an absence of intermodulation distortion. See *Methods (Technical Validation)* for details.

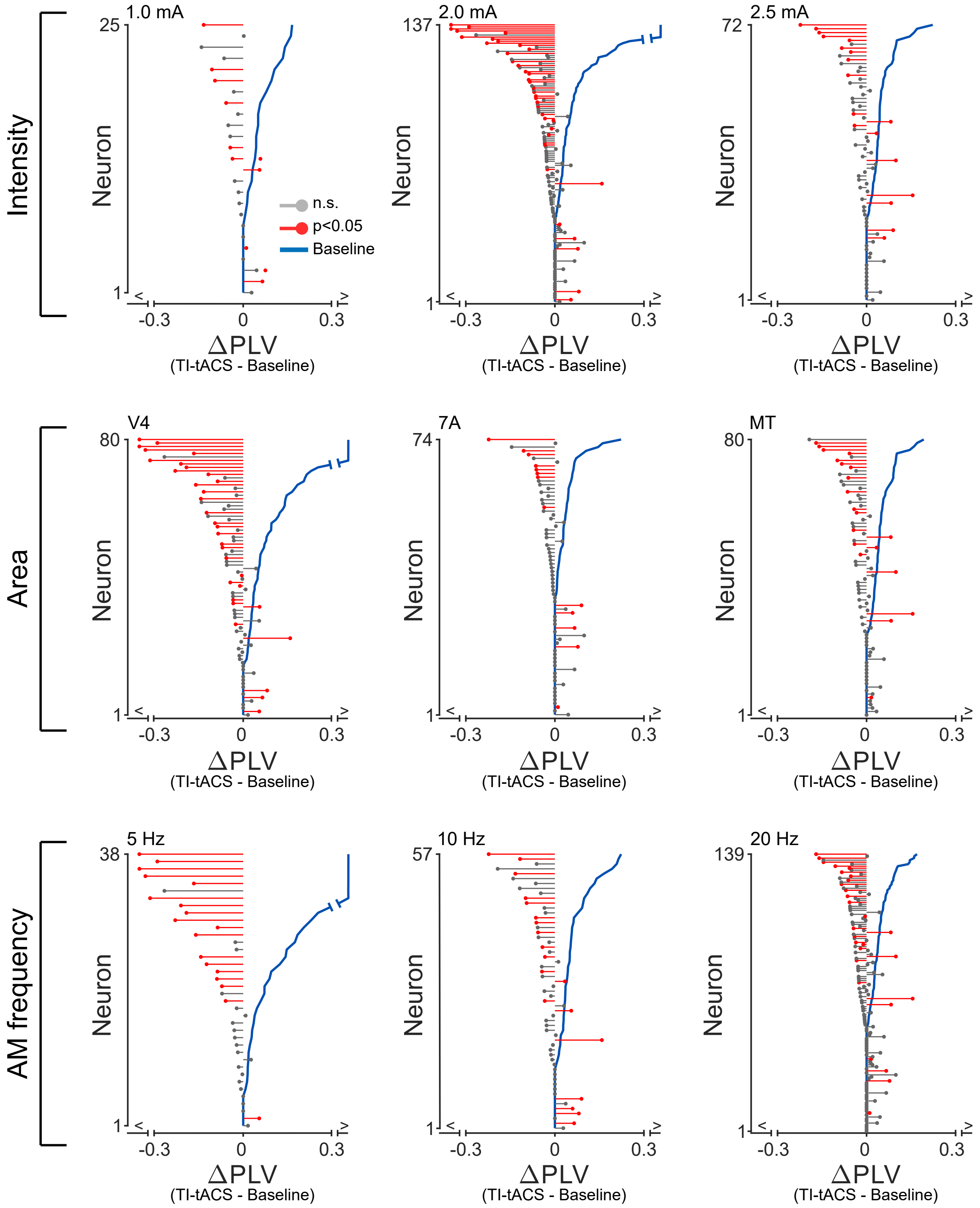


**Supplementary Figure 3: Entrainment effects, separated by stimulation amplitude, brain area and AM frequency**. Data is plotted in the same style as main Figure 3, except that a few extreme values are reported as < ±0.3 PLV or ΔPLV. See Main Figure 3 for exact values. As described in the main text, neither area (middle) nor AM frequency (bottom) were not significant predictors of ΔPLV. Instead, the baseline PLV (blue) and stimulus intensity (top) jointly determine the effects of subsequent stimulation on ΔPLV. The number of data points is shown on each vertical axis.

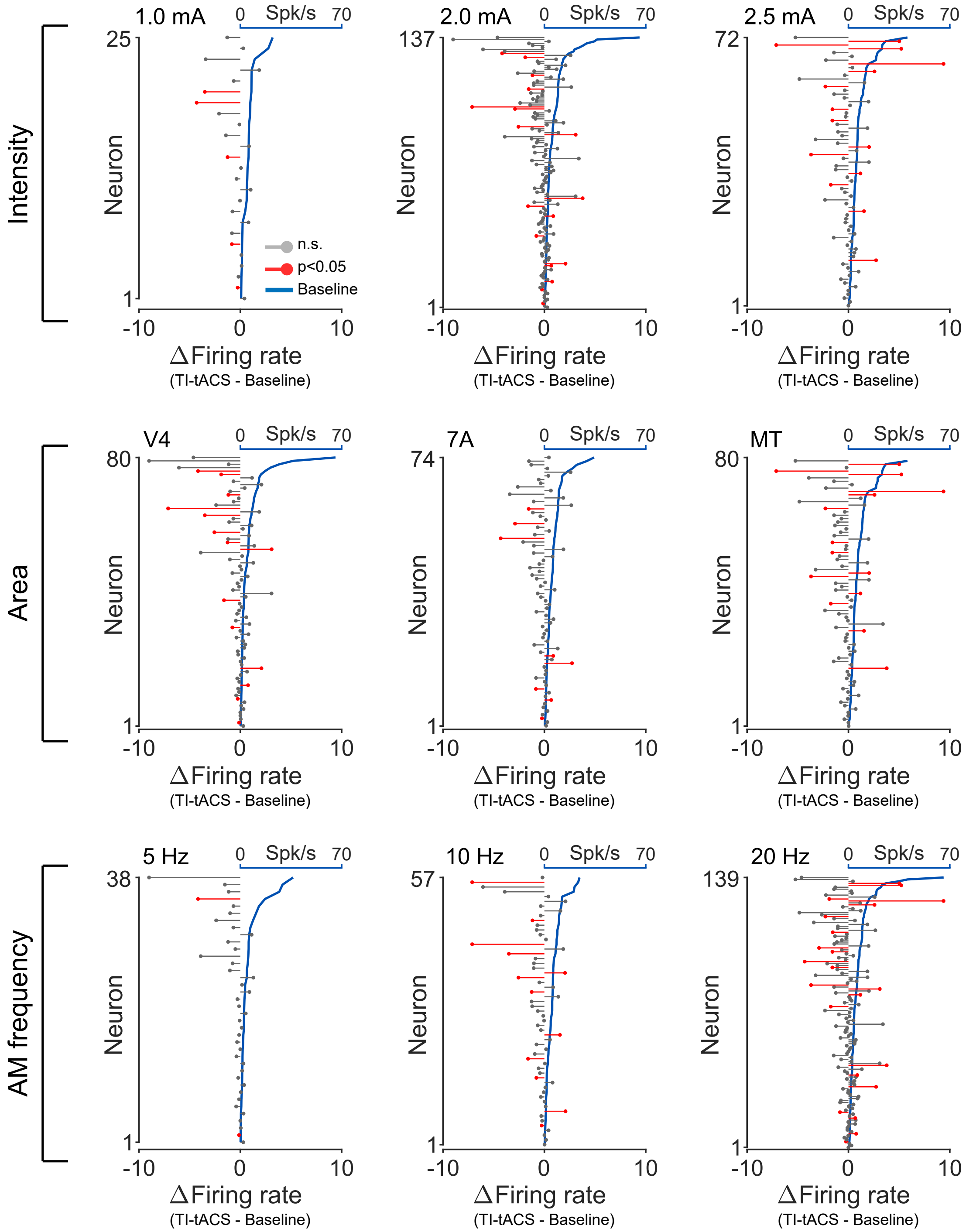


**Supplementary Figure 4: Rate effects, separated by stimulation amplitude, brain area and AM frequency**. Data is plotted in the same style as Supplementary Figure 3, except that the baseline values are shown on a separate scale (top of each graph). TI-tACS minimally affected firing rates.

Supplementary Table 1 – Stimulation current and predicted field strength used in human literature

| Paper | Stimulation current (mA) | Field strength (Predicted – V/m) |
|---|---|---|
| Violante et al. (2023) | ±2.0 (per pair); ±1.0 and ±3.0 | 0.4 V/m |
| Wessel et al. (2023) | ±0.5, ±1.0, ±1.5, ±2.0 (per pair) | 0.3 V/m |
| Ma et al. (2021) | ±1.0 (per pair) | - |
| Iszak et al. (2023) | ±3.4 (per pair) * | - |
| Zhu et al. (2022) | ±1.0 (per pair) | 0.5 V/m |
| Von Conta et al. (2022) | ±1.0 (per pair) | 0.2 V/m |
| Zhang et al. (2022) | ±1.0 (per pair) | - |

*Includes peripheral and cranial stimulation.

**Supplementary Table 1: Stimulation current and predicted field strength used in human literature.** Current intensities and (predicted) field strengths are in line with our experimental conditions, as shown in Figure 1 and Supplementary Figure 1.
