# Supplementary material for: Temporal interference stimulation disrupts spike timing in the primate brain
Source: Nat Commun. 2024 May 29;15:4558. doi: 10.1038/s41467-024-48962-2 (PMC11137077; doi:10.1038/s41467-024-48962-2)
Supplement: Supplementary file 3 — Reporting summary [file 41467_2024_48962_MOESM3_ESM.pdf]

nature portfolio

Corresponding author(s): Krause

Last updated by author(s): April 16, 2024

Reporting Summary

Nature Portfolio wishes to improve the reproducibility of the work that we publish. This form provides structure for consistency and transparency in reporting. For further information on Nature Portfolio policies, see our Editorial Policies and the Editorial Policy Checklist.

Statistics

For all statistical analyses, confirm that the following items are present in the figure legend, table legend, main text, or Methods section.

n/a	Confirmed	
☐	☒	The exact sample size (n) for each experimental group/condition, given as a discrete number and unit of measurement
☐	☒	A statement on whether measurements were taken from distinct samples or whether the same sample was measured repeatedly
☐	☒	The statistical test(s) used AND whether they are one- or two-sided<br>*Only common tests should be described solely by name; describe more complex techniques in the Methods section.*
☐	☒	A description of all covariates tested
☐	☒	A description of any assumptions or corrections, such as tests of normality and adjustment for multiple comparisons
☐	☒	A full description of the statistical parameters including central tendency (e.g. means) or other basic estimates (e.g. regression coefficient) AND variation (e.g. standard deviation) or associated estimates of uncertainty (e.g. confidence intervals)
☐	☒	For null hypothesis testing, the test statistic (e.g. F, t, r) with confidence intervals, effect sizes, degrees of freedom and P value noted<br>*Give P values as exact values whenever suitable.*
☒	☐	For Bayesian analysis, information on the choice of priors and Markov chain Monte Carlo settings
☒	☐	For hierarchical and complex designs, identification of the appropriate level for tests and full reporting of outcomes
☐	☒	Estimates of effect sizes (e.g. Cohen's d, Pearson's r), indicating how they were calculated

Our web collection on statistics for biologists contains articles on many of the points above.

Software and code

Policy information about availability of computer code

Data collection	Matlab (2016B), Psychtoolbox (3), Brainsight (2.5.1) , SimNIBS (3.2.6), 3DSlicer (4.10)
Data analysis	Matlab (2016B; 2020B),

For manuscripts utilizing custom algorithms or software that are central to the research but not yet described in published literature, software must be made available to editors and reviewers. We strongly encourage code deposition in a community repository (e.g. GitHub). See the Nature Portfolio guidelines for submitting code & software for further information.

Data

Policy information about availability of data

All manuscripts must include a data availability statement. This statement should provide the following information, where applicable:

- Accession codes, unique identifiers, or web links for publicly available datasets
- A description of any restrictions on data availability
- For clinical datasets or third party data, please ensure that the statement adheres to our policy

All data points supporting the findings of this study are available within the paper and its Supplementary Information. Owing to their size, the raw data are available upon request from the authors.

Research involving human participants, their data, or biological material

Policy information about studies with human participants or human data. See also policy information about sex, gender (identity/presentation), and sexual orientation and race, ethnicity and racism.

Reporting on sex and gender	n/a
Reporting on race, ethnicity, or other socially relevant groupings	n/a
Population characteristics	n/a
Recruitment	n/a
Ethics oversight	n/a

Note that full information on the approval of the study protocol must also be provided in the manuscript.

Field-specific reporting

Please select the one below that is the best fit for your research. If you are not sure, read the appropriate sections before making your selection.

[x] Life sciences [ ] Behavioural & social sciences [ ] Ecological, evolutionary & environmental sciences

For a reference copy of the document with all sections, see nature.com/documents/nr-reporting-summary-flat.pdf

Life sciences study design

All studies must disclose on these points even when the disclosure is negative.

Sample size	Data was collected from two macaque monkeys. However, the relevant unit of analysis here is the individual neurons (N=234), which were tested in a randomized, within-subject design. As described in the introduction, there was little agreement about the nature (let alone the magnitude) of the expected effect(s), making a formal power analysis impossible. We therefore used conditions similar to our prior work conventional tACS (e.g., Krause et al. 2019). See discussion in Methods.
Data exclusions	We included data from all neurons that were well-isolated from the background hash and remained present during the conditions of interest.
Replication	The methods section describes a number of technical controls intended to ensure that the results are not artifactual. Figures 4 compares results to a well-established technique (conventional tACS); Figure 5 and 6 investigate potential differences between tACS and TI.
Randomization	Neither animals nor neurons were assigned to a particular group; instead, each neuron was tested in both conditions, which were presented in a pseudorandomized order.
Blinding	Experimenters were not blind to the specific conditions. It would have been technically infeasible to blind ourselves to the conditions while monitoring signals for quality and potential artifacts.

Reporting for specific materials, systems and methods

We require information from authors about some types of materials, experimental systems and methods used in many studies. Here, indicate whether each material, system or method listed is relevant to your study. If you are not sure if a list item applies to your research, read the appropriate section before selecting a response.

Materials & experimental systems

n/a	Involved in the study	
x		Antibodies
x		Eukaryotic cell lines
x		Palaeontology and archaeology
	x	Animals and other organisms
x		Clinical data
x		Dual use research of concern
x		Plants

Methods

n/a	Involved in the study	
x		ChIP-seq
x		Flow cytometry
	x	MRI-based neuroimaging

Animals and other research organisms

Policy information about studies involving animals; ARRIVE guidelines recommended for reporting animal research, and Sex and Gender in Research

Laboratory animals	Rhesus macaques (M. mulatta): 11 year old male and a 10 year old female.
Wild animals	The study did not involve wild animals.
Reporting on sex	Standard practice in non-human primate research is to use a bare minimum of animals (two). Our data was collected from one male and one female. However, we cannot, even in principle, disentangle sex differences from other individual differences, and therefore do not report any sex-specific information.
Field-collected samples	This study did not involve samples collected in the field.
Ethics oversight	The Montreal Neurological Institute Animal Care Committee: Animal Use Protocols #5031 and #1870

Note that full information on the approval of the study protocol must also be provided in the manuscript.

Plants

Seed stocks	N/A
Novel plant genotypes	N/A
Authentication	N/A

Magnetic resonance imaging

Experimental design

Design type	Anesthesia/resting state -- structural only
Design specifications	7-10 averages per animal/modality
Behavioral performance measures	None

Acquisition

Imaging type(s)	structural only
Field strength	3T
Sequence & imaging parameters	T1w MPRAGE: TR = 2300 ms and TE = 3.59 ms); T2w: TR = 2800 ms and TE = 489 ms. 0.57 mm isotropic voxels. FOV adapted to capture each animals' head.
Area of acquisition	Whole brain

Diffusion MRI ☐ Used ☒ Not used

Preprocessing

Preprocessing software	Individual averages were rigid-body aligned and averaged in 3DSlicer
Normalization	Scans were used for experimental planning, and so normalization was not required.
Normalization template	Data were not normalized.
Noise and artifact removal	Slow head motion corrected by aligning individual averages.
Volume censoring	Artifacts clearly outside the head (e.g., phase wrap-around) were manually masked for alignment

Statistical modeling & inference

Model type and settings	N/A
Effect(s) tested	N/A

Specify type of analysis: [x] Whole brain [ ] ROI-based [ ] Both

Statistic type for inference (See Eklund et al. 2016)	N/A
Correction	N/A

Models & analysis

n/a	Involved in the study
[x]	[ ] Functional and/or effective connectivity
[x]	[ ] Graph analysis
[x]	[ ] Multivariate modeling or predictive analysis
